# Temperature-triggered chemical switching growth of in-plane and vertically stacked graphene-boron nitride heterostructures

**DOI:** 10.1038/ncomms7835

**Published:** 2015-04-14

**Authors:** Teng Gao, Xiuju Song, Huiwen Du, Yufeng Nie, Yubin Chen, Qingqing Ji, Jingyu Sun, Yanlian Yang, Yanfeng Zhang, Zhongfan Liu

**Affiliations:** 1 Center for Nanochemistry (CNC), Beijing Science and Engineering Center for Low-Dimensional Carbon Materials, Beijing National Laboratory for Molecular Sciences, College of Chemistry and Molecular Engineering, Peking University, Beijing 100871, China; 2 National Center for Nanoscience and Technology, Beijing 100190, China; 3 Department of Materials Science and Engineering, College of Engineering, Peking University, Beijing 100871, China

## Abstract

In-plane and vertically stacked heterostructures of graphene and hexagonal boron nitride (*h*-BN-G and G/*h*-BN, respectively) are both recent focuses of graphene research. However, targeted synthesis of either heterostructure remains a challenge. Here, via chemical vapour deposition and using benzoic acid precursor, we have achieved the selective growth of *h*-BN-G and G/*h*-BN through a temperature-triggered switching reaction. The perfect in-plane *h*-BN-G is characterized by scanning tunnelling microscopy (STM), showing atomically patched graphene and *h*-BN with typical zigzag edges. In contrast, the vertical alignment of G/*h*-BN is confirmed by unique lattice-mismatch-induced moiré patterns in high-resolution STM images, and two sets of aligned selected area electron diffraction spots, both suggesting a van der Waals epitaxial mechanism. The present work demonstrates the chemical designability of growth process for controlled synthesis of graphene and *h*-BN heterostructures. With practical scalability, high uniformity and quality, our approach will promote the development of graphene-based electronics and optoelectronics.

## Abstract (web summary)

Targeted synthesis of vertically stacked graphene (G) and hexagonal boron nitride (h-BN) heterostructures remains a challenge. Here, the authors achieve the selective, CVD growth of h-BN-G and G/h-BN through a temperature-triggered switching reaction.

## Graphical abstract

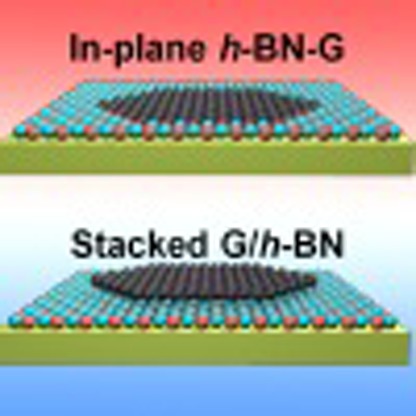

Graphene (G), a single atomic layer of carbon, has attracted considerable attention since its isolation^1^ in 2004, owing to its unique electrical and optical properties^2^,^3^. Hexagonal boron nitride (*h*-BN), a structural analogue of graphene, is an insulator (with a typical band gap of 5.8 eV^4^,^5^) possessing high in-plane mechanical strength and good chemical inertness even at elevated temperatures^6^,^7^. Recent work has focused on the development of both in-plane (*h*-BN-G) and stacked (G/*h*-BN) heterostructures containing graphene and *h*-BN, in the interest of combining their complementary properties for use in a broad range of applications. Varying the concentration of *h*-BN in *h*-BN-G is a promising method to tune the graphene band gap^8^,^9^,^10^. Synthesizing versatile *h*-BN-G is very likely be used in the construction of atomically thin integrated circuits in next-generation graphene devices^11^,^12^. Moreover, many efforts have been made to study the unique interface between *h*-BN and G in the hybridized atomic layer^13^,^14^,^15^,^16^. Meanwhile, G/*h*-BN has been exploited to fabricate high-performance graphene-based transistors using *h*-BN as a gate insulator, owing to its atomically flat and dangling-bond-free surfaces, which ensure that no charge trapping occurs at the G/*h*-BN interface. The stacked structure of G/*h*-BN allows for the easy observation of several unique physical phenomena such as the Hofstadter butterfly effect^17^,^18^,^19^.

The technical challenge is to develop an effective synthetic route for these hybrid material systems, which determines how far we can go for practical applications. Recent efforts have been focused on the chemical vapour deposition (CVD) technique. Of particular importance is those by Ajayan and coworkers, who demonstrated the synthesis of randomly distributed *h*-BN-G heterostructures on copper foils by simultaneously introducing carbon and ammonia borane precursors in the growth process^8^. Further follow-up studies reported the patterned graphene islands and ribbons embedded into *h*-BN layers by applying the so-called etching-regrowth methods^11^,^12^. Meanwhile, Zhang and colleagues reported the CVD synthesis of G/*h*-BN, accomplished by directly growing graphene on exfoliated *h*-BN. In this case, plasma-enhanced CVD process was needed due to the weak catalytic activity of *h*-BN^20^. Furthermore, Kong^21^ and Lee^22^ developed a two-step CVD method and obtained the G/*h*-BN stacks by sequentially growing *h*-BN and graphene on Cu foils. However, the unavoidable etching of the pre-grown *h*-BN layer in the second growth step results in both in-plane and stacked heterostructures simultaneously, leading to severe degradation of G/*h*-BN device performance. The lack of stacking registry control in the currently available synthetic strategies usually bring about graphene sheets randomly oriented with regard to *h*-BN, hindering the exploration of the unique quantum phenomenon induced by minor interlayer rotation^17^,^18^,^19^.

In the present study, we develop a novel temperature-triggered reaction route in CVD process to achieve the selective growth of *h*-BN-G and G/*h*-BN heterostructures on Cu foils, relying on a high carbon/hydrogen ratio molecule of benzoic acid as carbon source. Using atomically resolved scanning tunnelling microscopy (STM) and other methods, we systematically investigate the in-plane continuity and stacking configuration between graphene and *h*-BN for the synthesized *h*-BN-G and G/*h*-BN, respectively. Our work demonstrates the chemical designability of CVD process for deliberate control of graphene and *h*-BN heterostructures, offering a potential pathway for fabricating high-performance graphene-based electronic devices.

## Results

### Growth of *h*-BN-G and G/*h*-BN heterostructures

**Figure 1:**
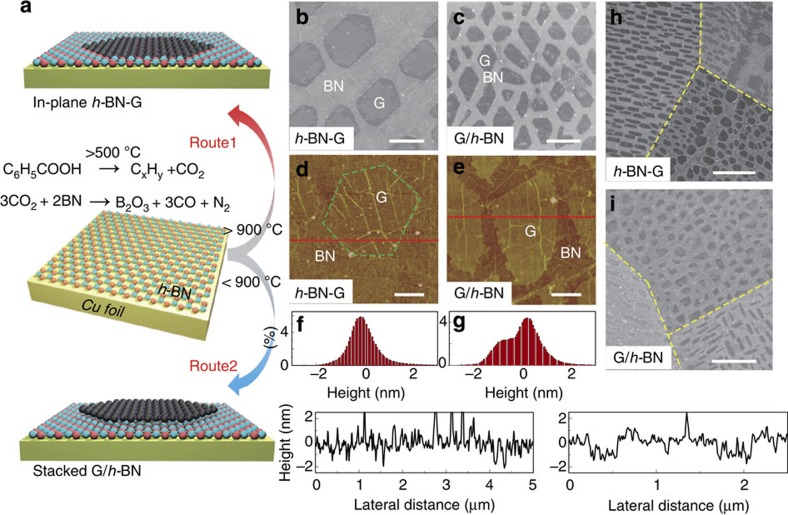
Growth of in-plane and vertically stacked graphene and boron nitride heterostructures. (**a**) Schematic illustration of the temperature-triggered switching growth between in-plane *h*-BN-G and stacked G/*h*-BN heterostructures, defined as Route 1 and Route 2, respectively. (**b**) SEM image of a sample synthesized by Route 1, showing discrete hexagonal graphene islands embedded in *h*-BN. (**c**) SEM image of a sample synthesized by Route 2, showing triangular and rhomboidal graphene islands grown on *h*-BN. The scale bars in **b** and **c** are 2 μm. (**d**,**e**) AFM height images of *h*-BN-G and G/*h*-BN, respectively, after being transferred onto 300-nm-thick SiO_2_/Si substrates, with corresponding height histograms and line sections shown in (**f**,**g**). The scale bars in **d** and **e** are 1 and 0.5 μm, respectively. (**h**,**i**) SEM images showing the facet-dependent growth behaviour of *h*-BN-G and G/*h*-BN, respectively. The scale bars are 10 and 5 μm, in **h** and **i**.

Figure 1a illustrates the temperature-triggered switching growth strategy of in-plane and stacked graphene and *h*-BN heterostructures. This process relies on the unique feature of the benzoic acid carbon source, which can decompose into carbon dioxide (CO_2_) and various hydrocarbon species on pyrolysis above 500 °C^23^. A complete *h*-BN monolayer is grown on a Cu foil by CVD, which serves as the first step of the whole process. On one hand, when the growth temperature for graphene exceeds 900 °C (Route 1) in the second step, the etching reaction of *h*-BN by CO_2_ is activated. As a result, the released CO_2_ partially etches away the *h*-BN monolayer, and the exposed Cu surface catalyses the subsequent graphene growth, leading to the formation of in-plane *h*-BN-G heterostructures. Note that, the by-products of the etching reaction can all be vaporized during the CVD process and can therefore be pumped away with the aid of the carrier gas. For confirming the etching reaction of *h*-BN with CO_2_ and the critical etching temperature, additional experiments were conducted for monolayer *h*-BN on both Cu foils and SiO_2_/Si substrates by directly using CO_2_ gas. The obtained results clearly indicate the occurrence of etching reaction at 900 °C, in which the underlying Cu plays an important role in inducing the etching reaction (See Supplementary Fig. 1 and Supplementary Note 1.). Similar experiments were also done by directly using CO gas, it is found that CO was nonreactive with *h*-BN at both 850 °C and 900 °C (See Supplementary Fig. 2 and Supplementary Note 2.). On the other hand, when the growth temperature is below 900 °C (Route 2), CO_2_ does not react with *h*-BN so that the *h*-BN etching reaction is deactivated. The hydrocarbon fragments from benzoic acid pyrolysis gradually grow into graphene islands on the intact *h*-BN layer, leading to the formation of stacked G/*h*-BN heterostructure. In brief, the temperature-dependent *h*-BN etching reaction serves as a switch to determine the types of heterostructures to be synthesized. With other growth parameters remaining unchanged, the critical temperature for reaction and growth switching is ∼900 °C.

The monolayer nature of the pre-grown *h*-BN layer was confirmed by atomic force microscopy (AFM) height analysis after the sample was transferred onto SiO_2_/Si (See Supplementary Fig. 3 and Supplementary Note 3.). The scanning electron microscopy (SEM) image of a sample synthesized by Route 1, shown in Fig. 1b, reveals discrete, darker hexagonal graphene islands embedded in monolayer *h*-BN. AFM was employed to confirm the patching nature of the sample by determining the height difference of the two regions again. These measurements were performed after transferring the sample onto a flat SiO_2_/Si substrate (Fig. 1d). Obviously, the corresponding height histogram over the whole layer presents a single-peak distribution (Fig. 1f), indicating the formation of in-plane patching structure of graphene and *h*-BN in the second growth step, owing to the concomitant BN etching reaction. The SEM image of a sample synthesized by Route 2 (Fig. 1c), using a growth temperature of 850 °C, shows that the graphene islands formed in the second growth step are much more irregular than that synthesized by Route 1. AFM height statistics exhibit a two-peaked distribution with a height difference of ∼0.93 nm between graphene and *h*-BN regions (Fig. 1e,g), suggesting the formation of monolayer graphene islands sitting on the predeposited *h*-BN. This increased apparent height of graphene on *h*-BN may arise from the possible water or atmospheric species intercalation, as mentioned by the previous works^3^,^21^,^24^. Interestingly, for a given Cu crystalline grain, the orientation of the graphene islands is almost parallel to each other for both *h*-BN-G and G/*h*-BN (Fig. 1h,i). Moreover, for both heterostructures, different Cu grains, mostly Cu(111) and (100) crystalline facets in the present experimental conditions, generate graphene islands of different shapes. These observations suggest that the underlying Cu crystalline facets strongly affect the crystalline orientations of *h*-BN and subsequently grown graphene islands for both heterostructures.

### Spectroscopic characteristics of *h*-BN-G and G/*h*-BN

**Figure 2:**
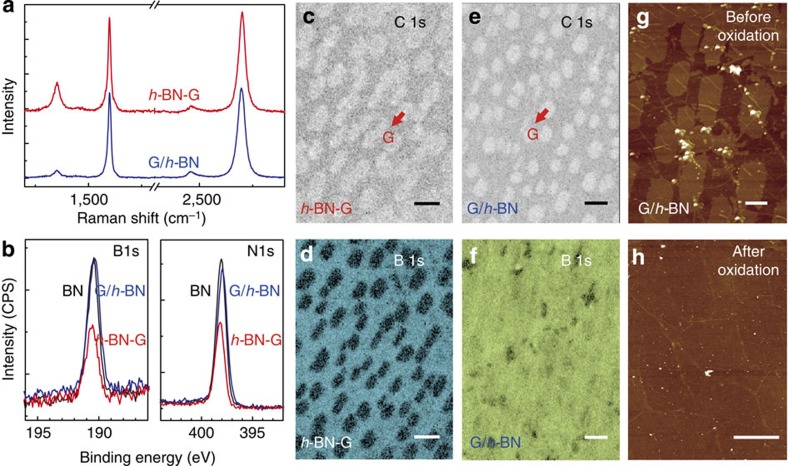
Spectroscopic characteristics of *h*-BN-G and G/*h*-BN. (**a**) Raman spectra of graphene regions in *h*-BN-G and G/*h*-BN. (**b**) B 1s and N 1s core-level XPS spectra of pure *h*-BN, in-plane *h*-BN-G and stacked G/*h*-BN on Cu foils. (**c**,**d**) AES C 1s and B 1s surface mapping of *h*-BN-G, showing the separating feature of graphene and *h*-BN regions in element distribution. (**e**,**f**) AES C 1s and B 1s surface mapping of G/*h*-BN, suggesting the presence of *h*-BN under graphene islands. The scale bars in **c**–**f** are 2 μm. (**g**) AFM height image of G/*h*-BN film on a 300 nm SiO_2_/Si substrate. (**h**) AFM height image of a G/*h*-BN film after etching at 600 °C under atmospheric conditions. The scale bars are 1 μm in **g** and **h**.

Raman spectroscopy provides further detailed information on the film structure and quality of samples after transferred onto SiO_2_/Si substrates. Single-point Raman spectra measured at the graphene regions of both *h*-BN-G and G/*h*-BN heterostructures are shown in Fig. 2a, which exhibit sharp (FWHM=∼33 cm^−1^) and symmetric 2D peaks (at ∼2,680 cm^−1^) with a 2D/G intensity ratio exceeding 1, suggesting its monolayer nature. Corresponding Raman mapping data also convinces the thickness uniformity and the monolayer feature of graphene regions in the two heterostructures (See Supplementary Fig. 4 and Supplementary Note 4.). However, the D peak (∼1,353 cm^−1^) intensity of the graphene islands in *h*-BN-G is much larger than that of G/*h*-BN. This could be attributed to either the limited lattice substitution of graphene by B or N atoms introduced from the etching-patching growth process, or the small domain sizes of graphene and *h*-BN^25^.

In addition, the G peak position in Raman spectra shows distinct difference between the two heterostructures. In *h*-BN-G, the G peak is located at ∼1,600 cm^−1^, which is remarkably blue-shifted as compared with pristine CVD graphene on SiO_2_/Si substrates (1,585–1,590 cm^−1^). Although the graphene in *h*-BN-G may have been slightly doped by boron or nitrogen during the etching growth process, no more than 10 cm^−1^ blue or red shift of G peak in Raman spectra can be induced as previously reported^26^,^27^. This significant blue shift could be attributed to the *p*-doping effect of SiO_2_ or the adsorbed water layer between graphene and the hydrophilic substrate^28^,^29^,^30^. In contrast, the Raman signal from the graphene region in *h*-BN/G agrees well with that of pristine CVD graphene, with the G peak located between 1,585 and 1,590 cm^−1^. This is attributed to the clean interface formed between *h*-BN and graphene in G/*h*-BN, which is likely not contaminated during the wet-transfer process. These observations provide additional evidence of the monolayer nature of *h*-BN-G and stacked bilayer nature of G/*h*-BN.

X-ray photoelectron spectroscopy (XPS) and Auger electron spectroscopy (AES) were utilized for elemental analysis to obtain the chemical bonding information and spatial distribution of specific elements in the two-step CVD-grown heterostructures. Figure 2b shows the B 1 s and N 1 s core-level photoelectron spectra of pure *h*-BN monolayer (upper black curve), *h*-BN-G (bottom red curve) and G/*h*-BN (blue curve) on Cu foils. All spectra were normalized by the Cu 2p 3/2 peak intensities. For both *h*-BN-G and G/*h*-BN, the B 1s peak is centred at ∼190.9 eV while the N 1s peak is located at ∼398.2 eV, both of which are very close to those of pure *h*-BN. Note that, the intensities of both B 1s and N 1s peaks in G/*h*-BN are almost identical to those of pure *h*-BN, while they are significantly decreased in *h*-BN-G. This can be easily explained by their different film structures. Considering the XPS analysis depth, the entire *h*-BN layer underneath graphene in G/*h*-BN can be ‘seen', and nearly the same XPS signal as that in pure *h*-BN appears. Obviously, in the *h*-BN-G film, the surface coverage decrease of *h*-BN results in the intensity decrease of XPS signal.

Figure 2c,e exhibits the AES spatial distributions of C 1s. The carbon-rich regions in *h*-BN-G and G/*h*-BN are present as similar isolated islands, consistent with the discrete island-shaped graphene SEM images shown in Fig. 1b,c. However, B 1s surface AES maps of *h*-BN-G and G/*h*-BN shown in Fig. 2d,f are significantly different. Apparently, the darker regions in Fig. 2d, corresponding to graphene islands, contain almost no boron signal, indicative of the isolated feature of carbon-rich and boron-rich regions. In contrast, the B 1s map in Fig. 2f shows homogeneous contrast distribution throughout the detection area, suggesting that the pre-grown *h*-BN layer remains intact.

From the above investigations, we can achieve the stacked G/*h*-BN structure by low-temperature two-step growth route, with the monolayer graphene islands being stacked on the pre-grown *h*-BN monolayer, forming a structural analogue of bilayer graphene. It is an interesting issue to understand the stacking order of two layers, which is directly related to the growth mechanism. In fact, recent studies utilizing ^13^C isotope labelling techniques prove that the second layer growth most likely occurs underneath the existing layer in the CVD bilayer graphene growth process on Cu foil^31^,^32^. To the best of our knowledge, no relevant experimental work has been reported for the CVD-grown G/*h*-BN heterostructures. We designed an in-air oxidation experiment to assign the stacking order of G/*h*-BN as shown in Fig. 2g,h. A G/*h*-BN film with a submonolayer graphene coverage was transferred onto a 300-nm-thick SiO_2_/Si substrate. Notably, the graphene flakes can be easily distinguishable from that of *h*-BN, as is shown in the AFM height image in Fig. 2g. The G/*h*-BN sample was then annealed in air at 600 °C for 2 h, sufficient for completely etching away all the graphene flakes (AFM image in Fig. 2h) according to the previous work^33^. The chemical inertness of *h*-BN has been observed previously, which can effectively prevent the oxidation of the underlying substrates^34^,^35^,^36^. Therefore, our oxidation result strongly supports that the graphene flakes are sitting on the *h*-BN monolayer, which is completely different from the bilayer graphene. XPS depth analysis also serves as the additional evidence for supporting this conclusion (See Supplementary Fig. 5 and Supplementary Note 5.). Overall, the second-step graphene growth occurs on the *h*-BN monolayer, forming a graphene/*h*-BN/Cu foil structure.

### Differences in surface potential between *h*-BN-G and G/*h*-BN

**Figure 3:**
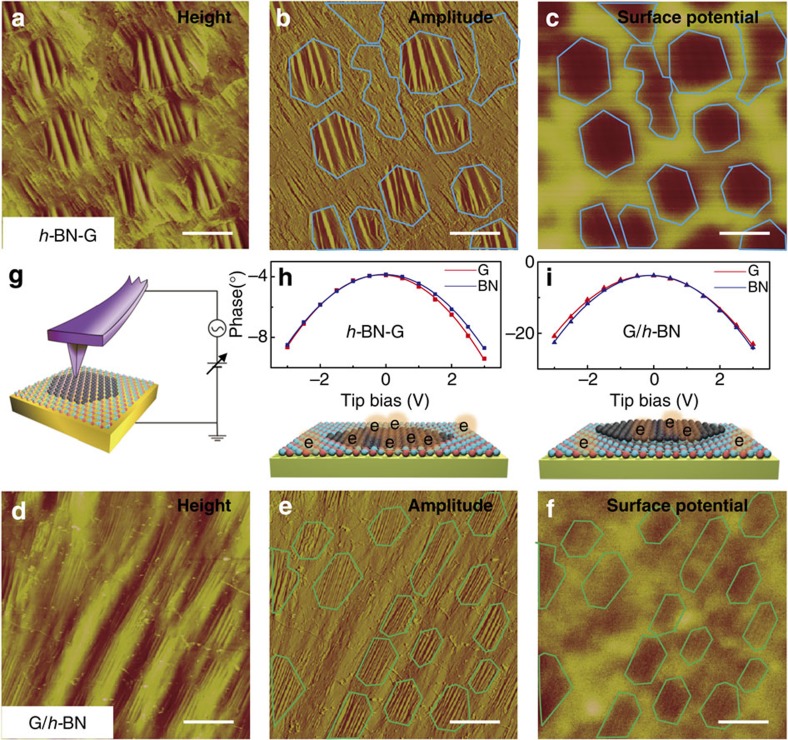
Differences in surface potential and work function between *h*-BN-G and G/*h*-BN. AFM height (**a**) and amplitude (**b**) images and EFM surface potential image (**c**) for *h*-BN-G on Cu foils, showing contrast for graphene regions, as marked by hexagonal shapes. Similarly, AFM height (**d**) and amplitude (**e**) images and EFM surface potential (**f**) image for G/*h*-BN on Cu foils, showing minor differences between graphene and *h*-BN regions. (**g**) Schematic diagram of the EFM measurement. (**h**,**i**) EFM phase plotted as a function of tip bias voltage for graphene (red) and BN (blue) in *h*-BN-G and G/*h*-BN, respectively. The scale bars in **a**–**f** are 2 μm.

Electrostatic force microscopy (EFM) has been proven effective to evaluate the layer number of 2D materials^37^,^38^. By measuring the surface potential or work function, we may distinguish the in-plane *h*-BN-G from stacked G/*h*-BN and understand the details of interfacial charge transfer. In EFM measurements, two-pass lift mode was used with 0.1 V bias voltage applied between a conductive AFM tip and the sample. Figure 3a–c show the AFM height, amplitude and EFM surface potential images, respectively, of *h*-BN-G on Cu foils. Notably, graphene regions (labelled by light blue lines in Fig. 3b) are more corrugated than the surrounding *h*-BN. Similar phenomenon has been reported by Han *et al*.^15^ and Cho *et al*.^39^, which was attributed to the different thermal expansion effects of graphene, *h*-BN and Cu substrate. As seen from the surface potential image shown in Fig. 3c, the embedded graphene islands can be clearly distinguished from the surrounding *h*-BN. In this case, the surface potential of graphene region is ∼80 meV lower than that of *h*-BN. The situation for stacked G/*h*-BN film is highly different as shown in Fig. 3d–f. The graphene area exhibits only ∼20 meV decrease in surface potential as compared with that of the *h*-BN area. Note that the surface potential in EFM measurements (Fig. 3g) reflects the local surface charge distribution when a bias voltage is applied between the EFM probe and the sample. In the case of G/*h*-BN film, the presence of dielectric *h*-BN layer between graphene and Cu substrate will certainly screen the charging effect from Cu substrate, leading to smaller surface potential difference between the two kinds of regions (G/*h*-BN/Cu and *h*-BN/Cu) than that of a *h*-BN-G film (*h*-BN/Cu and G/Cu).

The work function differences between graphene and *h*-BN regions can be calculated by plotting the EFM phase as a function of the tip bias voltage for *h*-BN-G and G/*h*-BN, as shown in Fig. 3h,i. After parabolic fitting, work function difference was deduced from the peak shifts of the curves. For both *h*-BN-G and G/*h*-BN, graphene regions present a smaller work function than that of *h*-BN regions, with negative differences (−82 and −47 meV, respectively). This means that the graphene regions are electrostatically *n*-doped by the underlying Cu substrate, regardless of the presence of *h*-BN layer in between, consistent with the previous theoretical calculations^40^.

The catalytic activity of a catalyst is closely related to its electronic density of states near the Fermi level, which can also, to some extent, be reflected by the work function. From the chemical point of view, the catalytic activity of *h*-BN would be much poorer than that of Cu for the dehydrogenation of carbon precursors. In fact, when using partly covered *h*-BN on Cu foil as the substrate, the subsequent graphene growth primarily occurred on Cu surface rather than on *h*-BN (Shown in Supplementary Fig. 6 and Supplementary Note 6.), suggesting the catalytic activity difference of two surfaces. However, a recent work by Joshi *et al*.^41^ indicates that the *h*-BN-covered Cu(111) surface exhibits a similar surface electronic property to pristine Cu(111), possessing the periodic standing wave patterns characteristic of metals in differential conductance (*dI/dV*) maps. A comparative experiment was performed on the thick *h*-BN for graphene growth. It is found that only amorphous carbons were obtained as seen in Supplementary Fig. 7 (See Supplementary Note 7 for more details.). This would be explained by the synergistic catalysis effect of *h*-BN and Cu for the subsequent graphene growth in the *h*-BN/Cu bilayer structure. Recent experimental and theoretical works^22^,^42^ also support our observation. Undoubtedly, the perfect lattice matching between graphene and *h*-BN would greatly facilitate the epitaxial growth of graphene on the *h*-BN/Cu surface. Moreover, the visible small Raman D peak at ∼1,353 cm^−1^ in G/*h*-BN (Fig. 2a) may imply the reduced catalytic activity of such a system on the dissociation of carbon precursors.

### STM characterizations of *h*-BN-G and G/*h*-BN

**Figure 4:**
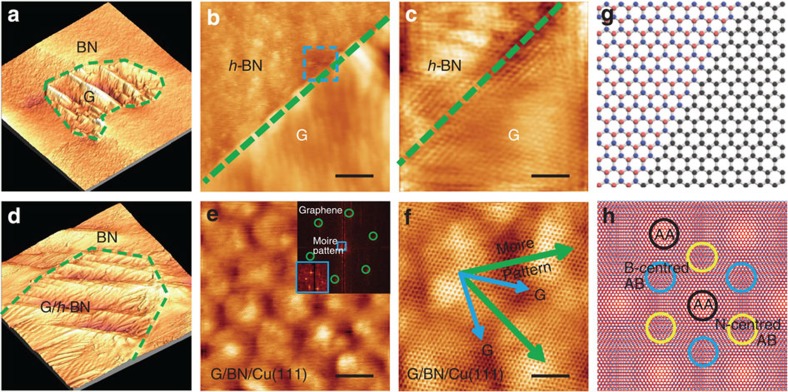
Atomically resolved STM characterizations of *h*-BN-G and G/*h*-BN. (**a**) STM image (800 nm × 800 nm) (−0.020 V, 1.061 nA) showing an island of graphene embedded in *h*-BN, with boundaries labelled by the green dashed line. (**b**) Enlarged STM image (−0.020 V, 1.061 nA) showing a sharp and continuous in-plane heterojunction between graphene and *h*-BN. The scale bar in **b** is 40 nm. (**c**) Zoom-in STM image (−0.002 V, 65.853 nA) of the region corresponding to the blue square in (**b**). The scale bar in **c** is 2 nm. (**d**) STM image (800 nm × 800 nm) (−0.007 V, 3.225 nA) showing a graphene island on *h*-BN, with boundaries labelled by the green dashed line. (**e**) STM image (−0.002 V, 7.850 nA) of G/*h*-BN showing the formation of a large moiré pattern due to the lattice mismatch between graphene and *h*-BN. The scale bar in **e** is 5 nm. The inset in (**e**) is a 2D-FFT image of the entire image. (**f**) Zoom-in STM image (−0.002 V, 10.785 nA) of the moiré pattern, with graphene lattice vectors aligned along blue arrows and moirés along green arrows. The scale bar in **f** is 3 nm. (**g**,**h**) Schematic atomic diagrams of in-plane patched graphene and *h*-BN with a zigzag interface and vertically-aligned G/*h*-BN with a large-period moiré formation.

STM has been employed recently to examine the stacking order of graphene and *h*-BN heterostructures^16^,^43^,^44^,^45^, which is also used here to give a straightforward evidence on the different configurations of our *h*-BN-G and G/*h*-BN heterostructures. Similar to the above EFM images, the regions of *h*-BN and graphene can be easily identified in STM images according to their different surface corrugations, with the graphene regions being more corrugated than that of *h*-BN (Fig. 4a,d). In the case of in-plane *h*-BN-G, as seen from the STM images captured along the patching boundary in Fig. 4b,c, the graphene region is resolved as much clearer atomic lattice than that of *h*-BN, and a perfect atomic scale in-plane patching can be evolved between graphene and *h*-BN. Of particular importance is the predominant zigzag-type interface patching structure, as schematically shown in Fig. 4g. This result is in good agreement with our previous work on the graphene patching growth into *h*-BN on the Rh(111) surface^14^. Note that, a preferred zigzag-type edge between graphene and *h*-BN is also reported on Cu foils recently^38^. The edge type was considered to depend on the specific growth condition. In contrast, for the G/*h*-BN system, the high-resolution STM images are characteristic of a large-scale moiré pattern as clearly seen in Fig. 4e,f and Supplementary Fig. 8 with a period of ∼7.8 nm. From the inserted 2D FFT image in Fig. 4e and the atomically resolved lattice in Fig. 4f, it is found that the angle between the moiré pattern and the graphene lattice vector equals almost 30°.

According to LeRoy and coworkers^46^ result, the period (*λ*) of a moiré pattern can be calculated using the following equation:


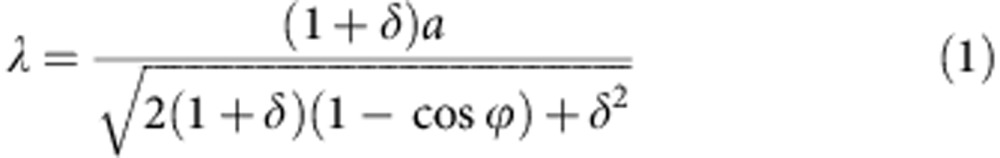


where *δ* and *ϕ* are the lattice mismatch and relative rotational angle between graphene and *h*-BN, respectively, and *a* is the graphene lattice constant. From the above equation, *λ* equals 12.9 nm when *ϕ* is set to 0°. The difference between the observed and theoretical periods is believed to arise from the perfect epitaxial growth mode of graphene on *h*-BN, which leads to three local stacking configurations, namely, AA stacking, nitrogen-centred and boron-centred AB stackings, respectively, as schematically illustrated in Fig. 4h. Assuming that graphene is well aligned with the underlying *h*-BN phase (without any misorientation), the three stacking configurations cannot be distinguished from each other in the STM image obtained directly on Cu, resulting in a change of the moiré period from 12.9 nm to 
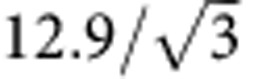
 (≈7.45) nm and a 30° rotation between the moiré pattern and the graphene lattice. This agrees well with our observations, also consistent with a recent result by Roth *et al*.^47^ about the stacked G/*h*-BN on Cu(111).

### Stacking geometry of G/*h*-BN

**Figure 5:**
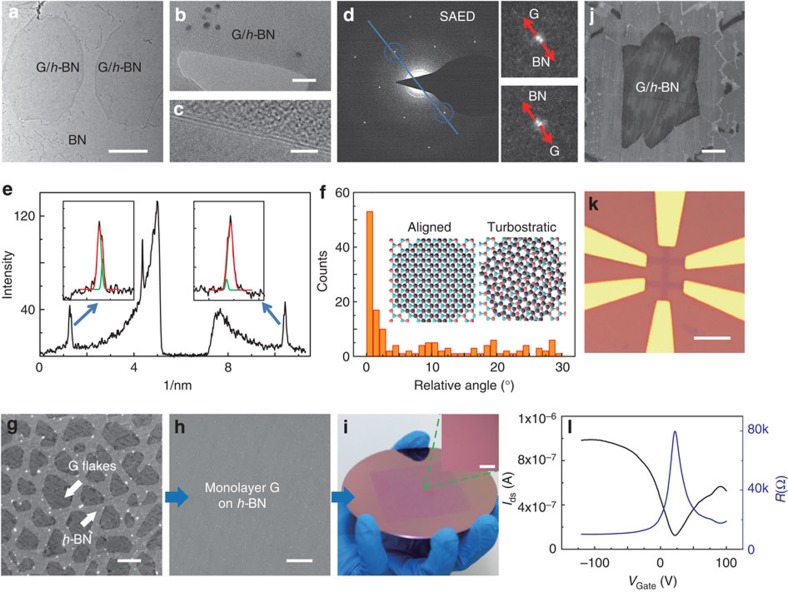
Stacking geometry of G/*h*-BN. (**a**) Large-scale TEM image of graphene islands on *h*-BN. (**b**,**c**) Magnified TEM images of (**a**) along the film edges. The scale bars in **a**–**c** are 1 μm, 20 and 2 nm, respectively. (**d**) SAED data demonstrating the aligned configuration of G/*h*-BN with the close-up views of the diffraction spots marked by circles. (**e**) Intensity profile along the blue line in (**d**) identifying the graphene and *h*-BN diffraction spots. (**f**) Stacking registry survey of G/*h*-BN, suggesting a well-aligned vertical stacking geometry. (**g**,**h**) SEM images of *h*-BN partially and fully covered by graphene, respectively. (**i**) Photograph of the wafer-scale G/*h*-BN film transferred onto SiO_2_/Si substrate with an OM image as an inset. (**j**) SEM image of large graphene domain grown on *h*-BN. The scale bars in **g**–**j** are 1, 2, 20 and 5 μm, respectively. (**k**) Optical microscope image of graphene back-gated field effect transistor (FET) fabricated on 300 nm SiO_2_/Si. The scale bar in **k** is 20 μm. (**l**) Device drain current (*I*_ds_) and resistance (*R*) versus gate voltage (*V*_gate_).

To confirm the stacking registry and twist angle between graphene and *h*-BN in the G/*h*-BN film on a large scale, high-resolution transmission electron microscopy (HR-TEM) combined with selected area electron diffraction (SAED) measurements were conducted. The TEM image of G/*h*-BN with submonolayer graphene coverage in Fig. 5a depicts two discrete graphene flakes showing higher contrast than the underlying *h*-BN. Figure 5b displays the TEM view of the G/*h*-BN film with full graphene coverage, where the edges of the sheet breakage allow for the direct identification of a bilayer. The HR-TEM image in Fig. 5c further confirms the bilayer nature and uniformity of G/*h*-BN. Shown in Fig. 5d is the SAED measurement of G/*h*-BN heterostructure, which exhibits a similar sixfold diffraction pattern. Close-up views of the regions identified by blue circles show two separated spots along the radial direction. The outer spot should correspond to the graphene lattice due to its relatively shorter lattice constant of 0.246 nm. Meanwhile, the intensity profiles along the radial direction of the SAED spots are plotted in Fig. 5e, where the peaks marked with blue arrows yield two components after Gaussian fitting. The ratio of the distance between the outer and inner peaks, representing the lattice parameter ratio between graphene and *h*-BN, is calculated to be 1.0166, consistent with the theoretical value (0.246/0.250=1.016).

To better understand the stacking registry of thus grown G/*h*-BN, a statistical analysis of the twist angles is given in Fig. 5f based on 182 surveyed SAED results. The histogram indicates that graphene is preferentially aligned with the crystalline direction of the underlying *h*-BN, following a van der Waals epitaxial mode.

For electronic applications, it is essential to achieve a continuous, large area and uniform growth of monolayer graphene on *h*-BN. As discussed above, at the initial epitaxial growth step of monolayer graphene on *h*-BN, discrete micrometer-scale flakes were usually evolved with relatively high nucleation density (Fig. 5g). By extending the growth time from 10 to 20 min, fully covered graphene on *h*-BN was obtained as seen in Fig. 5h. A 4-inch wafer-scale G/*h*-BN film was achieved in such a way, which can be easily transferred onto SiO_2_/Si substrates (Fig. 5i). By controlling the feeding rate of benzoic acid and folding a Cu foil into an envelope, the nucleation density of graphene on *h*-BN could be greatly decreased along with a drastic increase of the graphene domain size (∼20 μm) (SEM image in Fig. 5j). A field effect transistor (FET) was fabricated on the G/*h*-BN film transferred onto 300 nm SiO_2_/Si substrate using Au/Cr contacts (Fig. 5k). The carrier mobility at an estimated carrier density of 5 × 10^11^ cm^−2^, obtained from whole branch reaches a level of 15,000 cm^2^ V^−1^ s^−1^ (Fig. 5l) at room temperature, indicating the high quality of thus-grown G/*h*-BN samples (See Supplementary Note 8 for the details).

## Discussion

In summary, we have developed a novel temperature-triggered chemical switching growth technique for the selective CVD synthesis of in-plane and vertically stacked heterostructures of graphene and *h*-BN. The key of success is the control of *h*-BN etching reaction by CO_2_ molecules, generated from the decomposition of benzoic acid at high temperature. At a higher temperature over 900 °C, the etching reaction is triggered on, leading to the in-plane graphene patching growth into the pre-grown *h*-BN monolayer with a zigzag linking boundary. In contrast, at relatively low growth temperature, the etching reaction is turned off, which results in the epitaxial growth of graphene onto the *h*-BN monolayer, forming a stacked bilayer heterostructure. Our work demonstrates the chemical designability of CVD process for growing graphene and *h*-BN heterostructures in a well-controlled manner. With the practical scalability, high uniformity and crystalline quality, our approach will greatly promote the development of graphene-based electronics and optoelectronics.

## Methods

### Electropolishing process

The 25-μm Cu foil was purchased from Alfa Aesar China. Before growth, Cu foils were electropolished in a home-made plastic tank for 1 h, using a mixture of phosphoric acid (85%wt) and ethylene glycol (15%wt) as the electrolyte. In the electropolishing process, a 1.5 V DC voltage bias was applied between anodic Cu foil and cathodic Cu plate.

### Sample preparation

The pre-treated Cu foil was loaded into a 1-inch quartz tube and heated by a Thermal split tube furnace (Lindberg/Blue M HTF55347c) for graphene and *h*-BN growth. Using a rotary pump, a base pressure of 0.5 Pa can be achieved in our system. During growth, working pressure was held at 1.2 × 10^2^ Pa, either with or without the introduction of benzoic acid.

With respect to the in-plane *h*-BN-G growth, Cu foil was first heated from room temperature to 1,000 °C in 40 min, with 20 sccm (standard cubic centimetre per minute) H_2_ and 50 sccm Ar as carrier gas. Followed by 10 min annealing at 1,000 °C, ammonia borane, solid BN precursor, was introduced into furnace using a heating belt to grow monolayer *h*-BN (usually for 10 min). After that, furnace temperature was set to 900–920 °C for graphene growth, using solid benzoic acid as precursor, which was sublimated by a heating belt at ∼100 °C.

Growth parameters of stacked G/*h*-BN were identical with that of *h*-BN-G described above, except that the furnace temperature was set to 850 °C during graphene growth process.

### Characterization

Characterizations were performed with SEM (Hitachi S4800), AFM (Veeco Nanoscope IIIa, tapping mode), Raman (Horiba, LabRAM HR-800, 514.5 nm, 2.41 eV), XPS (Kratos Analytical AXIS-Ultra with monochromatic Al Kα X-ray), AES (PHI Quantera SXM), STM (Omicron UHV-VT-SPM-MBE System) and TEM (FEI Tecnai G2 F20, acceleration voltage 200 kV; FEI Tecnai G2 T20, acceleration voltage 200 kV).

### Transport measurement

After G/*h*-BN samples being transferred onto a 300 nm SiO_2_/Si substrate, the back-gated graphene FET was fabricated on a single graphene domain. With the help of electron beam lithography, graphene channels were patterned and (100 nm/5 nm) Au/Cr metal contacts were thermally evaporated. The transport measurements were performed at room temperature using a Keithley 4200-SCS semiconductor characterization system, equipped with a four-probe station. During measurements, a 0.1-V DC bias was applied between source and drain, with a linear sweep gate voltage from −100 to 80 V applied on back gate.

## Author contributions

Z.L. and Y.Z. conceived and supervised the research project. T.G. developed and conducted the growth of graphene and boron nitride heterostructures, with X.S.'s assistance. H.D. and Y.Y. performed the EFM characterization. T.G., X.S., Y.N, Y.C., Q.J. and J.S. carried out SEM, OM, Raman, XPS, AFM, STM, TEM and transport measurements. T.G., Z.L., Y.Z., J.S. and X.S. co-wrote the manuscript and all authors contributed to the critical discussions and the revision of the final manuscript accordingly.

## Additional information

**How to cite this article:** Gao, T. *et al*. Temperature-triggered chemical switching growth of in-plane and vertically stacked graphene-boron nitride heterostructures. *Nat. Commun*. 6:6835 doi: 10.1038/ncomms7835 (2015).

## Supplementary Material

Supplementary Information Supplementary Figures 1-8, Supplementary Notes 1-8 and Supplementary References
