# Supplementary material for: Temperature-triggered chemical switching growth of in-plane and vertically stacked graphene-boron nitride heterostructures
Source: Nat Commun. 2015 Apr 14;6:6835. doi: 10.1038/ncomms7835 (PMC4403442; doi:10.1038/ncomms7835)
Supplement: Supplementary Information — Supplementary Figures 1-8, Supplementary Notes 1-8 and Supplementary References [file ncomms7835-s1.pdf]

Supplementary Information

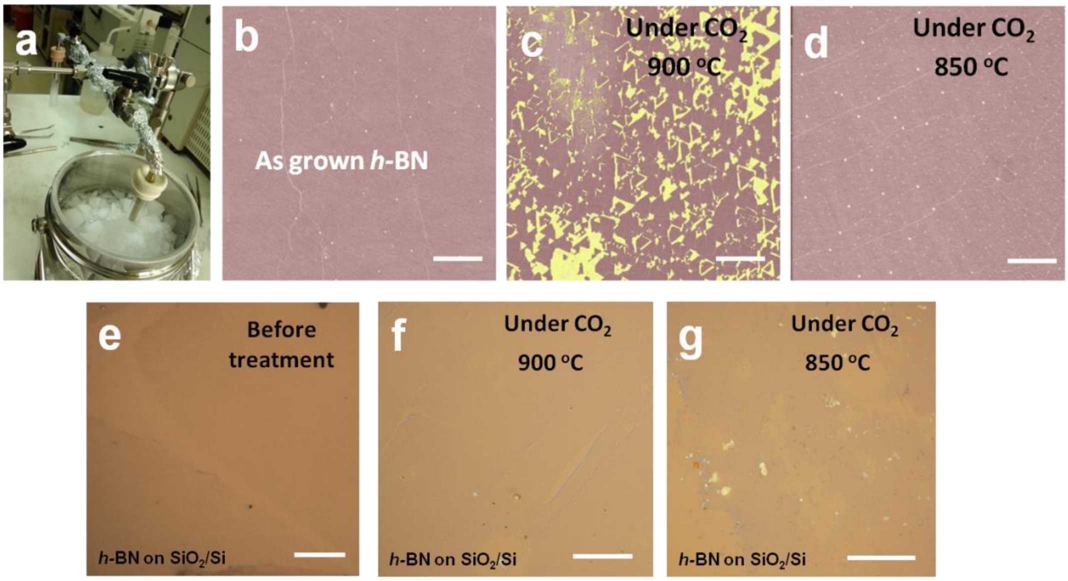

Supplementary Figure 1 | SEM and OM images of etching results of *h*-BN on Cu foil and SiO_2/Si substrates by CO_2 at 900 °C and 850 °C (**a**) Photograph of the experimental setup for inletting CO_2 into the CVD furnace. (**b-d**) False-color SEM images of as-grown *h*-BN on Cu foils, after reacted with CO_2 at 900 °C and 850 °C, respectively. The scale bars in **b-d** are 2 μm. (**e**) OM image of transferred *h*-BN on an 80 nm SiO_2/Si substrate. (**f, g**) OM images of *h*-BN on 80 nm SiO_2/Si substrates after being treated under CO_2 flow at 900 °C and 850 °C, respectively. The scale bars in **e-f** are 25 μm.

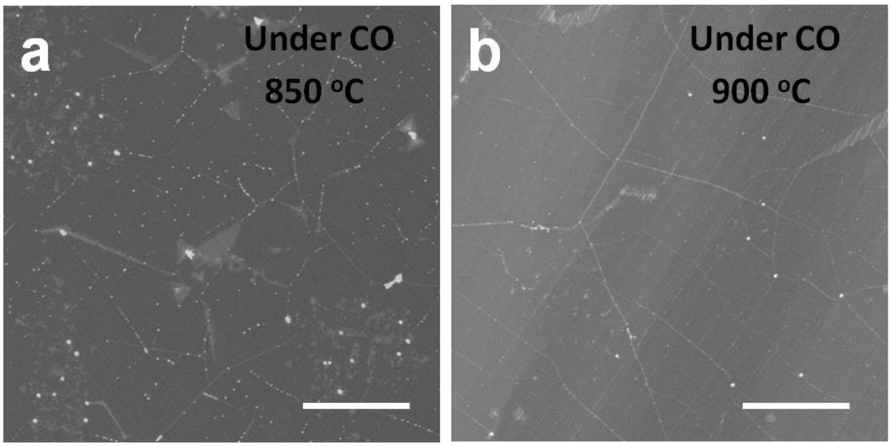

Supplementary Figure 2 | SEM images of the etching results of *h*-BN on Cu foils by the flow of CO at 900 °C and 850 °C SEM images of *h*-BN on Cu foils after being treated with CO at 850 °C (**a**) and 900 °C (**b**), respectively. The scale bars are 25 μm.

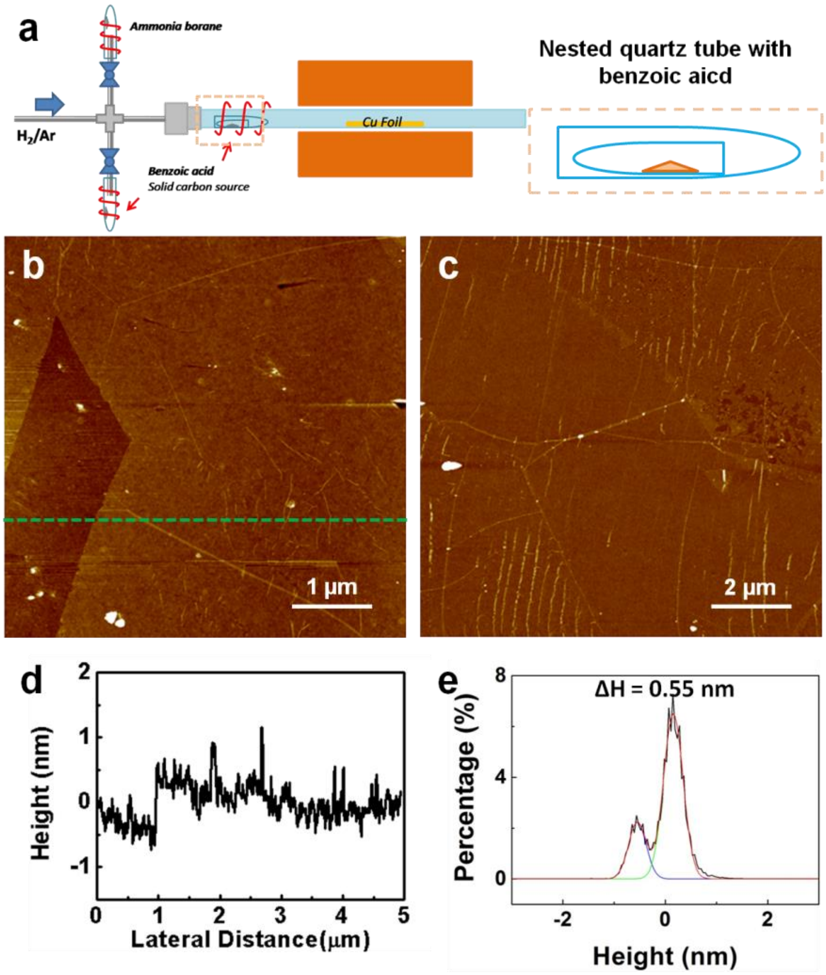

Supplementary Figure 3 | Experimental setup schematic diagram and the AFM height analysis of produced monolayer *h*-BN film. (**a**) Schematic diagram of the LPCVD setup. (**b, c**) AFM height images of partially-covered and fully-covered monolayer *h*-BN on SiO_2/Si substrates. The scale bars in **b** and **d** are 1 and 2 μm. (**d**) Height profile extracted along the green dashed line in (**b**). (**e**) Height histogram of the whole image of (**b**) suggesting the monolayer nature of the pre-deposited *h*-BN.

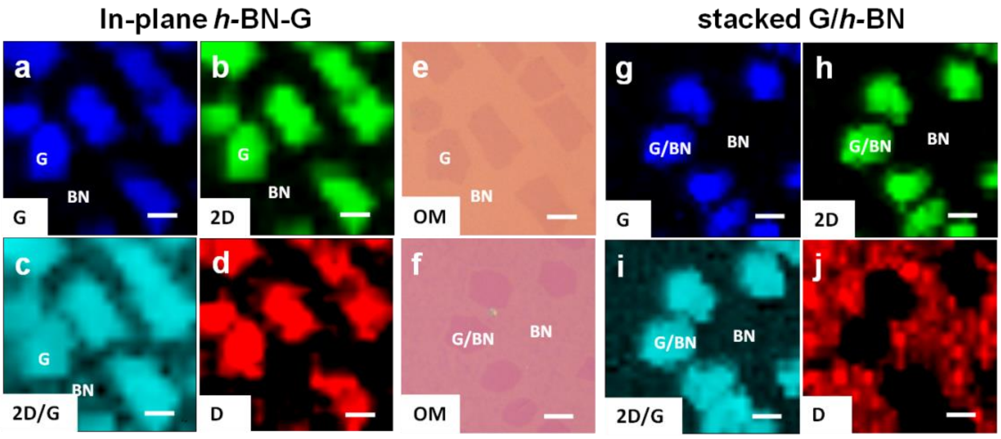

Supplementary Figure 4 | Raman mapping of *h*-BN-G and G/*h*-BN. Raman G peak, 2D peak, 2D/G ratio and D peak intensity mapping images of in-plane *h*-BN-G (**a-d**), and vertically stacked G/*h*-BN (**g-j**). Corresponding OM images of in-plane *h*-BN-G (**e**) and stacked G/*h*-BN (**f**). The scale bars are 5 μm.

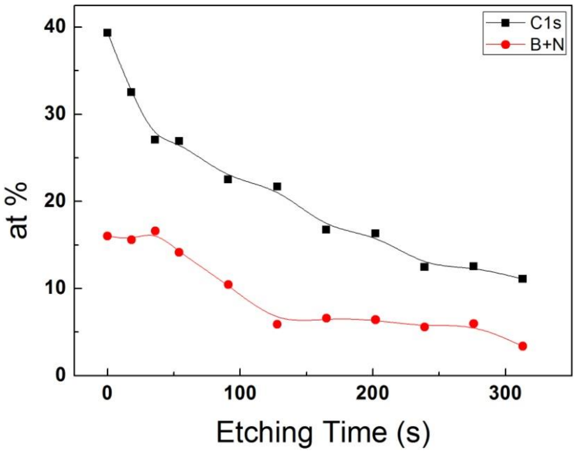

Supplementary Figure 5 | XPS depth analysis of G/*h*-BN sample on Cu foil. Atomic concentration curves of carbon (solid square) and boron with nitrogen (solid circle) versus etching time obtained from XPS depth analysis.

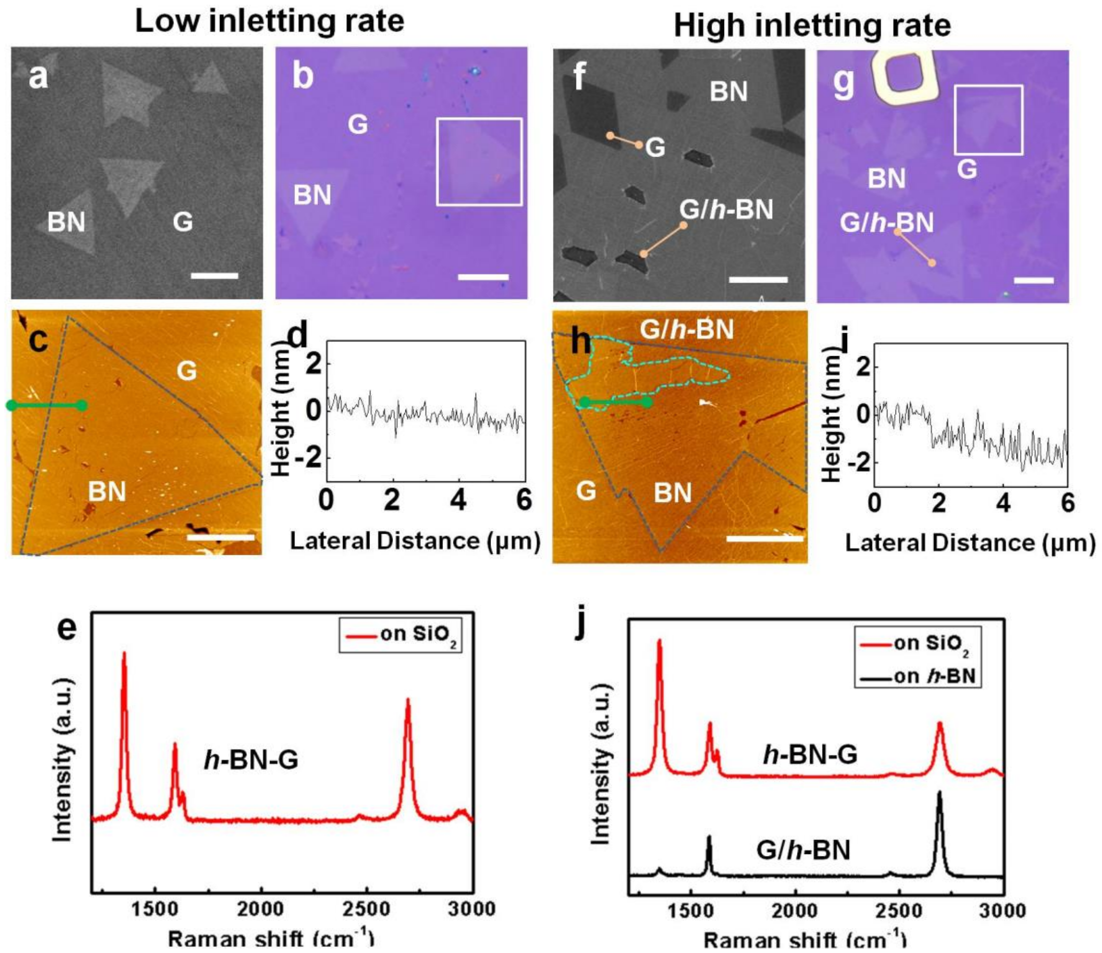

Supplementary Figure 6 | The influence of the inlet rate of the carbon precursor. (**a**) SEM image showing the patching of graphene onto individual *h*-BN islands on Cu foils. (**b**) Corresponding OM image after sample transfer onto 300 nm SiO_2/Si substrate. (**c**) AFM height image of the sample region labeled by a white square in (**b**). (**d**) Corresponding line profile along the green line in (**c**). (**e**) Raman spectrum of graphene around the *h*-BN island. Under high benzoic acid inletting rate, (**f**) SEM image showing the growth of graphene on incomplete *h*-BN layer on Cu foils. (**g**) OM image after transfer onto a 300 nm SiO_2/Si substrate. (**h**) AFM height image of the sample region labeled by a white square in (**g**). (**i**) Corresponding line profile along the green line in (**h**). (**j**) Raman spectrum of graphene around and above *h*-BN. The scale bars in **a, b, f** and **g** are 10 μm, and the scale bars in **c** and **h** are 5 μm.

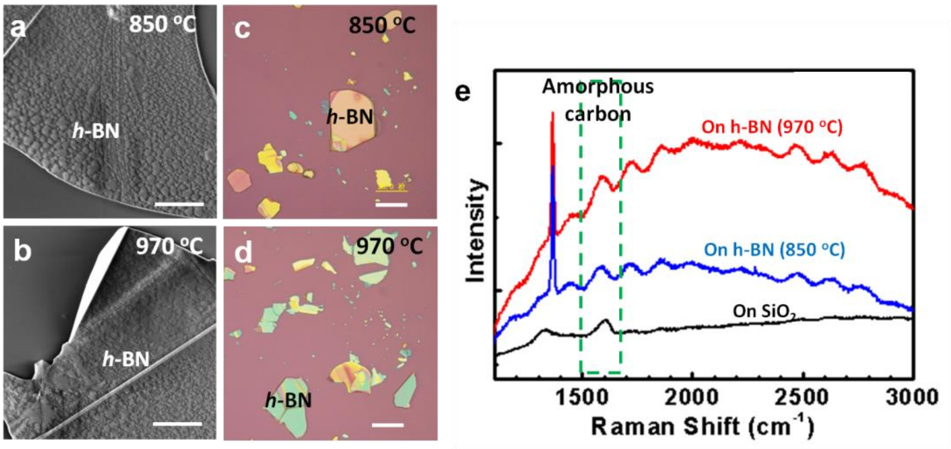

Supplementary Figure 7 | Graphene growth results on bulk *h*-BN using benzoic acid precursor. SEM images of bulk *h*-BN after growth process at 850 ^{o}C (**a**) and 900 ^{o}C (**b**). The scale bars are 4 μm. OM images of bulk *h*-BN after growth process at 850 ^{o}C (**c**) and 900 ^{o}C (**d**). The scale bars in **c** and **d** are 20 μm. (**e**) Corresponding Raman spectra.

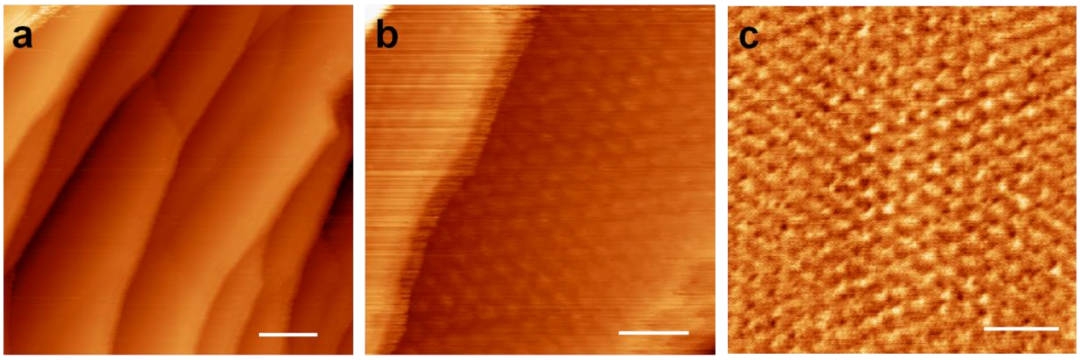

Supplementary Figure 8 | STM images of large-area uniform graphene moiré patterns. (**a**) STM image (-0.002 V, 7.850 nA) of G/*h*-BN on Cu foils. (**b, c**) Zoom-in views of large-area uniform moiré patterns evolved from the different lattice parameters of graphene and *h*-BN. The scale bar is 50 nm in **a**, 20 nm in **b** and **c**.

Supplementary Note 1. Etching reaction of *h*-BN on Cu foil and SiO_2/Si substrates by CO_2 at 900 °C and 850 °C.

The temperature triggered etching reaction of *h*-BN with CO_2 is considered to serve as a switch in the synthesis of in-plane or vertically stacked graphene and *h*-BN heterostructures on Cu foils. For investigating the etching capability of CO_2, a series of control experiments were carried out using drikold as CO_2 source, under the identical conditions with that in the CVD growth of the heterostructures (20 sccm H_2 and 50 sccm Ar as carrier gas, working pressure at 1.2×10^{-2} Pa). As shown in Supplementary **Fig. 1a**, drikold was loaded into a quartz tube, cooled in the drikold/acetone bath, and sublimated with a very slow rate. A ball valve was utilized to control the introduction of CO_2 into the CVD system.

Supplementary **Fig. 1b** shows a typical SEM image of as-grown monolayer *h*-BN on Cu foils. Except for some striped line-shape contrasts (corresponding to *h*-BN winkles), the film surface is characterized with a uniform contrast. As shown in Supplementary **Fig. 1c**, after its reaction with CO_2 at 900 °C, monolayer *h*-BN was partially etched leading to the exposure of basal Cu regions, which usually present brighter contrasts than the neighboring *h*-BN covered areas. However, the monolayer *h*-BN on Cu foils remained intact after its reaction with CO_2 at 850 °C (Supplementary **Fig. 1d**). This comparative investigation indicates that the temperature triggered etching reaction should occur at a critical temperature of 900 °C.

For evaluating the influence of the underlying Cu foil during the etching process, transferred CVD *h*-BN samples on 80 nm SiO_2/Si substrates (Supplementary **Fig. 1e**) were also treated with CO_2 at 900 °C and 850 °C, respectively. As shown in Supplementary **Fig. 1f** and **1g**, the *h*-BN samples remained intact after being treated with CO_2 at both 900 °C and 850 °C. These results indicate that the *h*-BN samples on 80 nm SiO_2/Si substrates are inert to CO_2 at 900 °C and 850 °C. Based on the above observations, it can be inferred that the underlying Cu plays an important role in assisting the etching reaction of *h*-BN with CO_2 at 900 °C.

Supplementary Note 2. The etching tendentiousness of *h*-BN on Cu foils by the flow of CO at 900 °C and 850 °C.

According to the chemical reaction $3CO_2 + 2BN \rightarrow B_2O_3 + 3CO + N_2$, B_2O_3, CO, and N_2 are the main products. The generated CO may also serve as an etchant of *h*-BN. In order to evaluate the etching ability of CO, a series of control experiments were carried out on *h*-BN/Cu at 850 and 900 ^oC, under 5 sccm CO flow (with 20 sccm H_2 and 50 sccm Ar as carrier gases). The concentration of employed CO in the test experiment should be much higher than that of the by-product from the above chemical reaction in the normal growth condition.

As clearly shown in Supplementary **Figs. 2a** and **b**, *h*-BN samples on Cu foils remain intact under CO flow at both 850 ^oC and 900 ^oC. In this regard, the CO by-product (from the chemical reaction of CO_2 and *h*-BN) should not react with pre-existing *h*-BN in the subsequent graphene-growth step for the growth of heterostructures.

Supplementary Note 3. Experimental setup for *h*-BN growth on Cu foils and the thickness characterization of produced *h*-BN.

It has been reported that, both monolayer and multilayer *h*-BN can be synthesized on Cu foils, by using ammonia borane (BH_3NH_3) as precursor[1]. In our experiments, by optimizing the LPCVD experimental setup and growth parameters, we have realized the synthesis of strictly monolayer *h*-BN on Cu foils. The flow rate of the ammonia borane was well controlled by our unique design of the source container (two nested quartz tubes), along with a very accurate control of the heating temperature (with a fluctuation of ~ 0.5^o C) (Supplementary **Fig. 3a**).

In order to determine the layer number, AFM height analysis was performed on the transferred *h*-BN samples. The AFM images in Supplementary **Figs. 3b and c** are the partially-covered and fully-covered *h*-BN layers on SiO_2/Si. Corresponding height profile obtained from the marked line in Supplementary **Fig. 3d** shows a height of ~0.7 nm with regard to the substrate, in good agreement with the value for monolayer *h*-BN on SiO_2/Si. Furthermore, the accurate height difference between *h*-BN and substrate can be deduced from the histogram analysis in Supplementary **Fig. 3e**, which presents ~0.71 nm height difference between the two fitted peaks, suggestive of the monolayer nature of *h*-BN on Cu foils.

Supplementary Note 4. Two dimensional (2D) Raman mapping of in-plane *h*-BN-G and vertically stacked G/*h*-BN.

In order to show the crystal quality and thickness uniformity of graphene in the two types of heterostructures, 2D Raman mapping measurements were performed on the transferred samples. As shown from the OM images in Supplementary **Fig. 4e** and **f**, in-plane *h*-BN-G and stacked G/*h*-BN samples were transferred onto 300 nm SiO_2/Si. Herein, graphene regions are imaged as discrete islands with uniform contrasts, which indicates tentatively their thickness uniformity.

Likewise, the G peak, 2D peak, 2D/G ratio, and D peak Raman mapping images in Supplementary **Fig. 4a-d, g-j** also display rather uniform contrasts over the graphene regions, further confirming the high thickness uniformity, as well as the monolayer feature of the embedded graphene flakes inside *h*-BN, and the stacked G on *h*-BN.

Note that, the D peak intensity mapping of graphene regions (Supplementary **Fig. 4d**) in *h*-BN-G usually present bright contrasts, which is different from that of G/*h*-BN showing dark contrasts (Supplementary **Fig. 4j**). This might imply that the graphene regions in *h*-BN-G should have more defects than that of G/*h*-BN, arising from rough edge structures or the doping effects by N or B atoms during CVD growth.

However, due to our limited Raman excitation laser beam (514 nm), it is very hard to acquire a high signal-to-noise ratio of the typical vibration mode of *h*-BN (located at ca. 1368 cm^{-1}), typical for monolayer *h*-BN. Additionally, the signal of *h*-BN and the D peak of graphene presents a considerable overlap. The two factors make Raman mapping (at D peak or at 1368 cm^{-1}) not a suitable method for identifying the different configurations of the two heterostructures.

Supplementary Note 5. XPS depth analysis.

For the purpose of confirming the stacking geometry, XPS depth analysis was performed on the as-grown G/*h*-BN samples on Cu foils. The XPS instrument (Axis Ultra, Kratos Analytical Ltd.) we used in this work is equipped with a monochromatic Al Kα X-ray source ($h\nu$ = 1486.7eV). The analysis depth is less than 10 nm, which makes XPS a surface-sensitive characterization method.

In order to figure out whether the upper graphene can attenuate the photoelectron from *h*-BN or not, we had a careful check on the database of electron inelastic mean free path. According to our XPS data, the binding energy for B 1s and N 1s was measured to be 190.9 eV and 398.2 eV, respectively. Therefore, the kinetic energy of the photoelectron generated from B and N should be (1486.7 – 190.9 – φ) eV and (1486.7 – 398.2 – φ) eV, respectively, where φ is related to the work function (depending on both spectrometer and the material (with the value of only few eV)). Accordingly, the value of the kinetic energy of the photoelectron is estimated to be over 1 keV, which can give an estimated 2 nm mean free path in graphite. Considering the thickness of graphene is only ~0.34 nm, the attenuation factor/effect of the monolayer graphene could be neglected, considering that the thickness of graphene is only 0.34 nm.

In the depth analysis, an Ar^+ ion beam (with an energy of 4 kV) was used to bombard the surface, followed by standard XPS elemental analysis for measuring the atomic concentrations of C, B and N. As depicted in Supplementary **Fig. 5**, the concentration of C dramatically decreases with the increase of etching time at the initial stage (below 50 s), where the concentration of B and N remains unchanged. Note that, the drop of B, N concentration occurs only after the etching time going beyond 50 s, and the etching by Ar^+ bombardment started from the outer surface. The decrease of C concentration probably proves that graphene is located above *h*-BN. However, considering of the adventitious carbon contamination in XPS carbon spectra, XPS depth analysis can only serve as an indirect evidence for identifying the stacking order.

Supplementary Note 6. More proofs of the temperature trigged growth and the influence of the inlet rate of the carbon precursor.

For studying the possibility of the coexistence of in-plane *h*-BN-G and stacked G/*h*-BN during the secondary growth of graphene, incomplete *h*-BN/Cu samples was chosen as the ideal platform, which is composed of both exposed Cu and *h*-BN regions. Since graphene growth is very sensitive to the concentration of carbon precursor, different sublimating temperatures (85 ^{o}C and 110 ^{o}C) of benzoic acid were selected to achieve either relatively high or low inlet rates of carbon precursors.

As shown in the SEM image (Supplementary **Fig 6a**), under a low benzoic acid inlet rate, graphene growth only occurs around the triangle *h*-BN islands, right on the exposed Cu surface instead of on the *h*-BN surface. However, under a high benzoic acid inletting rate, graphene flakes is observed not only around *h*-BN islands, but also above the *h*-BN islands (shown as darker regions in the SEM image of Supplementary **Fig. 6f**).

After the sample transfer onto 300 nm SiO_2/Si, OM image in Supplementary **Fig. 6b** clearly shows that graphene is fully patched around *h*-BN under a low benzoic acid inlet rate. However,

using high inlet rate of benzoic acid, except for the patched graphene, some graphene flakes (labelled in Supplementary **Fig. 6g**) can also be observed on the *h*-BN island. This was also verified by AFM characterizations. Both the AFM image in Supplementary **Fig. 6c** and the related line profile in Supplementary **Fig. 6d** reveal that, graphene and the embedded *h*-BN share the same value of height, namely, they are of equal thickness. Additionally, the irregular shape graphene flake (Supplementary **Fig. 6h,** circled by dashed lines) is ~ 0.7 nm higher than the underneath *h*-BN island, as evidenced by the AFM height profile (Supplementary **Fig. 6j**). Besides, Raman spectra in Supplementary **Fig. 6e** and **j** further prove that graphene growth happens only on Cu surface when using a low benzoic acid inletting rate, and on both Cu and *h*-BN/Cu regions when using a relatively high inlet rate.

Briefly, in the second-step growth of graphene on the *h*-BN covered Cu substrate, graphene prefers to growth on the bare Cu regions than that of *h*-BN covered regions, as may be mediated by the higher catalytic abilities of Cu in terms of dehydrogenation than that of *h*-BN.

Supplementary Note 7. Attempt to grow graphene on bulk *h*-BN using benzoic acid precursor.

For understanding the different catalytic abilities of *h*-BN/Cu and bulk *h*-BN for subsequent graphene growth, control experiments were conducted for graphene growth at 850 ^{o}C and 970 ^{o}C, respectively. Note that, the bulk *h*-BN was prepared by scattering micrometer sized *h*-BN powder onto SiO_2/Si, which served as the growth substrate for graphene growth under the same synthetic conditions as that used in the heterostructure synthesis.

As observed from SEM images (Supplementary **Fig. 7a** and **b)**, randomly distributed "carbon" flakes are deposited on *h*-BN at both 850 and 970 ^{o}C. However, according to the Raman spectra (Supplementary **Fig. 7e**), no typical Raman vibrational mode (2D band) of graphene is observed on bare SiO_2, or on *h*-BN substrates after CVD growth at 850 ^{o}C or 970 ^{o}C, respectively. The peak occurred at ~1600 cm^{-1} (as marked by dashed rectangle) should indicate amorphous carbon rather than graphene formation on thick *h*-BN films[2]. Therefore, we come to a conclude that, without the catalytic effect from the underneath Cu foils, the growth of large-area, high-qulaity graphene on thick *h*-BN using our current routes is challenging.

Supplementary Note 8. Details of growth protocols and electronic measurements of large-domain G/*h*-BN.

It is well known to us that a high nucleation density of graphene on *h*-BN will degrade the quality of G/*h*-BN and further hinder the practical application in high performance electronics. Controlling the inlet rate of benzoic acid is hence the key factor to decrease the nucleation density in the second graphene growth procedure. Herein we in particular employed (i) a low sublimation temperature (85 ^{o}C) of carbon precursor and (ii) an envelope-folded Cu foil to prevent the exposed *h*-BN surfaces from the excessive deposition of carbon precursors, thus aiding the decrease of the nucleation density. As shown in **Fig. 5**, a nearly 20 um × 20 um graphene domain was obtained on *h*-BN.

After being transferred on to a 300 nm SiO_2/Si substrate, G/*h*-BN channels and (100 nm/5 nm) Au/Cr contacts were prepared using electron beam lithography (**Fig. 5)**. Further electronic measurements were carried out on these graphene field effect transistors, using 300 nm SiO_2 as the dielectric layer and Si as the back gate. The FET carrier mobility of graphene was calculated using the equation below:

$$\mu = m\frac{L}{W}\cdot\frac{1}{V_{DS}}\cdot\frac{1}{C_i}$$

where m is the slope of the linear part near charge neutrality point of graphene, L and W is the length and width of channel, V_{DS} is the bias voltage between source and drain contacts, C_i is the gate insulator capacitance per unit area, which is formed by 300 nm SiO_2 layer between graphene and back gate.

Based on the plotted I_{ds} versus V_{gate} curve (Fig. 5l), the value of our FET carrier mobility of G/*h*-BN could reach 15,000 $cm^2V^{-1}s^{-1}$. These transport measurements indicate that the monolayer underneath can sufficiently improve the performance of graphene electronics, considering that the value of FET carrier mobility of CVD-grown monolayer graphene is normally around 4,000 $cm^2V^{-1}s^{-1}$.

Supplementary References

1. Kim KK, *et al.* Synthesis of Monolayer Hexagonal Boron Nitride on Cu Foil Using Chemical Vapor Deposition. *Nano Letters* **12**, 161-166 (2011).

2. Ferrari A, Robertson J. Interpretation of Raman spectra of disordered and amorphous carbon. *Physical Review B* **61**, 14095-14107 (2000).
